# Editorial: Exploring the insect microbiome: The potential future role in biotechnology industry

**DOI:** 10.3389/fmicb.2022.1061360

**Published:** 2022-11-03

**Authors:** Yongjun Wei, Hongjie Li

**Affiliations:** ^1^School of Pharmaceutical Sciences, Zhengzhou University, Zhengzhou, China; ^2^Laboratory of Synthetic Biology, Zhengzhou University, Zhengzhou, China; ^3^Institute of Plant Virology, Ningbo University, Ningbo, China

**Keywords:** insects, microbiome, synthetic biology, gut microbiota, insect bacterial symbionts

Insects are the most diverse and the most ecologically dominant terrestrial animals since arthropods conquered the land (Engel, 2015). The success of insects is in the association with microbial mutualists, which consequently give rise to the emergence of even more diverse insect traits, such as feeding on recalcitrant plant material, protection against pathogens, and the enhancement of inter- and intraspecific communications. Insights into the insect microbiome represent a particularly promising source for bioactive compounds, natural products for environmentally-friendly pest control, and other industrial biotechnology applications (Liu et al., 2019). However, the diversity of insects and their microbial symbionts remains unexplored, and the identities and functions of enzymes/chemicals derived from insect symbionts have yet to be discovered and elucidated (Li et al., 2021b). Thus, exploring the untapped insect microbiome using multi-omics and synthetic biology technologies is of great interest.

In this Research Topic, some insect microbiota and their function were investigated. Insect gut microbiota play key roles in insect feeding and adaption to environments. Zhao et al. collected nine grasshopper species from Loess Plateau in North China and characterized the microbiota of these grasshoppers using 16S V3-V4 amplicons. They find that these nine grasshopper species only share 5.5% of their microbial species, showing different grasshoppers have specific gut microbiota. Li, Zhang, et al. used 16S V3-V4 amplicons to give insight into the microbiota of *Ectropis grisecens* that feed on tea leaves in central China. Moreover, they isolated one non-core gut bacteria *Bacillus* species, and this species could degrade fat bodies, which might help the host adapt to starvation. Calle-Tobón et al. fed *Aedes albopictus* with active and heat-inactivated serum, and they found that feeding mosquitoes with different food might affect mosquito gene expression and microbiome metabolism. However, feeding active and heat-inactivated serum has no effect on the virome core of the mosquito population.

Insect gut microbiota play a key role in plant virus transmission. Wu et al. summarized the impact of insect-symbiotic bacteria on plant virus transmission and found that insect-symbiotic bacteria might participate during viral circulation and viral vertical transmission. Moreover, the authors proposed that a future understanding of the interaction between viruses and insect-symbiotic microbes might develop a novel plant virus disease prevention strategy. Li, Guan, et al. reported a novel positive-sense single-stranded RNA virus in *Agrotis ipsilon* and its genomic characterization. This virus can stably infect another host, *Spodopterera frugiperda*, and confer deleterious effects on the infected host of *S. frugiperda*, showing this novel virus can transmit in the same moth family.

Microbial insect symbionts contain large amounts of unexplored biotechnological resources. Barcoto et al. compared the degradation of recalcitrant substrates of lignocellulose and plastic polymers and summarized the microorganisms and enzymes that participated in these two bioprocesses (Miao et al., 2022). Moreover, the authors reviewed insect fungiculture systems and their potential application in biotechnologies. Based on these lessons, the authors proposed the application of cutting-edge technologies to recover microorganisms and enzymes for plastic polymer degradation or other biotechnological applications from insect fungiculture (Jiang et al., 2021). *Cordyceps militaris* is an entomopathogenic fungus and can produce diverse biomolecules. Moreover, *C. militaris* is an edible mushroom that produces high-level cordycepin when grows on silkworm pupae. A hypoxic environment is beneficial for cordycepin production. Wang et al. overexpressed the sterol regulatory element binding proteins (SREBPs) in *C. militaris* and increased cordycepin more than two-fold, showing that SREBPs play a role in the growth and bioactive molecules synthesis in *C. militaris*. This study suggests that engineered microorganisms with desired characteristics can produce high-level biomolecules (Ma et al., 2021; Jiang et al., 2022).

The insect microbiome has diverse potential biotechnological applications, and systems biology and multi-omics can help give insight into the insect microbiome (Wei et al., 2018, 2020). This Research Topic not only covers the insect microbiome but also details several examples of the insect symbionts in the interaction of insect-insect microbiome-virus. Besides, the engineering of an insect-related fungus with synthetic biology strategies might lead to the high-level production of bioactive compounds (Li et al., 2021a). In all, we believe this Research Topic will eventually help discover novel bioactive compounds, enzymes, insect control strategies, and other biotechnological products from the insect microbiome.

## Author contributions

YW and HL conceived the study. YW wrote the manuscript. HL revised the manuscript. All authors revised the manuscript.

## Funding

This work was supported by grants from the National Key Research and Development Program of China (Grant Nos. 2019YFB1503904 and 2018YFA0900201) and the National Natural Science Foundation of China (Grant No. 32111530179).

## Conflict of interest

The authors declare that the research was conducted in the absence of any commercial or financial relationships that could be construed as a potential conflict of interest.

## Publisher's note

All claims expressed in this article are solely those of the authors and do not necessarily represent those of their affiliated organizations, or those of the publisher, the editors and the reviewers. Any product that may be evaluated in this article, or claim that may be made by its manufacturer, is not guaranteed or endorsed by the publisher.
